# A Case Report of CHEK2 and MUTYH Germline Mutations Associated With Cholangiocarcinoma in a Young Patient

**DOI:** 10.7759/cureus.22631

**Published:** 2022-02-26

**Authors:** Obaid Rehman, Bradley Sackfield, Viveksandeep Thoguluva Chandrasekar, Jorge Oliver, Ganesh Aswath

**Affiliations:** 1 Internal Medicine, Hamilton Medical Center, Dalton, USA; 2 Gastroenterology, Mayo Clinic in Arizona, Phoenix, USA

**Keywords:** mutyh mutation, chek2 mutation, extrahepatic, intrahepatic, cholangiocarcinoma

## Abstract

Cholangiocarcinoma (CCA) is a major cause of primary liver carcinoma and has been associated with the penetrance of several germline mutations. We present a 31-year-old female evaluated for left upper quadrant pain and abnormal liver function tests. Ultrasound revealed a nodule in the liver, and biopsy showed intrahepatic adenocarcinoma. Germline testing was positive for two mutations: c.1100delC and c.1227_1228dupGG on the CHEK2 gene and the MUTYH gene, respectively. The patient was started on chemotherapy and tolerated it well. We aimed to demonstrate an association between CHEK2 and MUTYH mutations with CCA and highlight the importance of genetic testing for at-risk patients.

## Introduction

Cholangiocarcinoma (CCA) is a leading cause of adenocarcinoma that arises in the glandular tissue of bile duct cells [1]. CCA can be classified as intrahepatic, extrahepatic, perihilar, or Klatskin tumors [1]. The overall incidence of CCA in the United States has steadily increased over the last 30 years with approximately 12,000 new cases diagnosed every year [2]. The majority of patients diagnosed with CCA present in the seventh decade of life, and males have a higher incidence compared to females with ratios of 2.5:1 [2]. Established risk factors include primary sclerosing cholangitis (PSC), parasitic infections, hepatolithiasis, biliary duct cysts, and various toxins [3,4]. Less established risk factors of CCA include ulcerative colitis, cirrhosis, hepatitis B/C virus, and germline mutations [3,4]. Germline mutations are primarily sporatic, occur randomly, and are inherited in a dominant pattern [5,6]. These mutations, however, do not always occur with equal probability, and a recessive inheritance pattern can occur [5,6]. CCA is frequently associated with mutations in TP53, IDH1, SMAD4, KRAS, ARID1A, BAP1, PBMR1, and CDKN2A [5,6]. Very few cases have linked CCA with CHEK2 and MUTYH mutations individually with no published cases of CCA with both mutations [5,6]. In this case report, we will explore the association of both CHEK2 and MUTYH mutations in a patient with CCA.

## Case presentation

A 31-year old woman presented to the gastroenterology office with worsening left upper quadrant pain and abnormal transaminases (Table 1). Past medical history was significant for benign essential hypertension, hyperlipidemia, chronic nonalcoholic fatty liver disease, obesity, polycystic ovarian syndrome (PCOS), asthma, and gastrointestinal reflux disease (GERD).

**Table 1:** Laboratory findings from the basic metabolic panel from March 2020 to August 2020 of patients with intrahepatic cholangiocarcinoma along with c.1100delC mutation on the CHEK2 gene and c.1227_1228dupGG on the MUTYH gene AST: Aspartate transaminase; ALT: alanine aminotransferase; Alk Phos: alkaline phosphatase; N/A*: not applicable.

Date	AST (IU/L); Ref. range: (10-30)	ALT (IU/L); Ref. range: (10-36)	Alk Phos (IU/L); Ref. range: (32-104)	Total Bilirubin (mg/dL); Ref. range: (0.2-1.0)	Albumin (g/dL); Ref. range: (3.5-5.0)
March 2020	45	100	53	0.5	4.5
April 2020	N/A*	N/A*	N/A*	N/A*	N/A*
May 2020	44	104	59	0.4	4.7
June 2020	52	108	61	0.5	4.7
July 2020	47	124	68	0.3	4.8
August 2020	45	134	67	0.6	4.4

**Figure 1:**
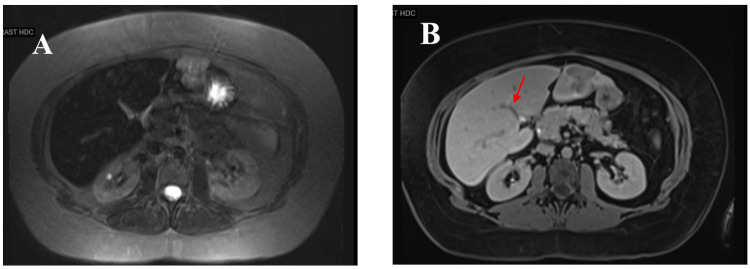
MRI of the liver left lobe of a 31-year-old woman showing a heterogenous, irregular, hyperintense mass on T2 in segment 3 (A) Five minutes after contrast injection, the lesions are not visible. (B) Twenty minutes after contrast injection, there are multiple, hyperintense lesions on T2 indicated by a red arrow, consistent with intrahepatic cholangiocarcinoma.

Ultrasound of the abdomen showed mild hepatomegaly with the liver measuring 18.2 cm as well as an area of slightly increased echogenicity and no focal lesions. The patient underwent magnetic resonance imaging (MRI) with and without contrast, which demonstrated a complex, hypoechoic, heterogeneous nodule along the tip of the left lobe in segment 3 measuring 3.5 cm x 3.4 cm x 3.1 cm. The mass was T2 hyperintense with areas of hepatic steatosis indicated by a red arrow in Figure 1, Panel B.

**Figure 2:**
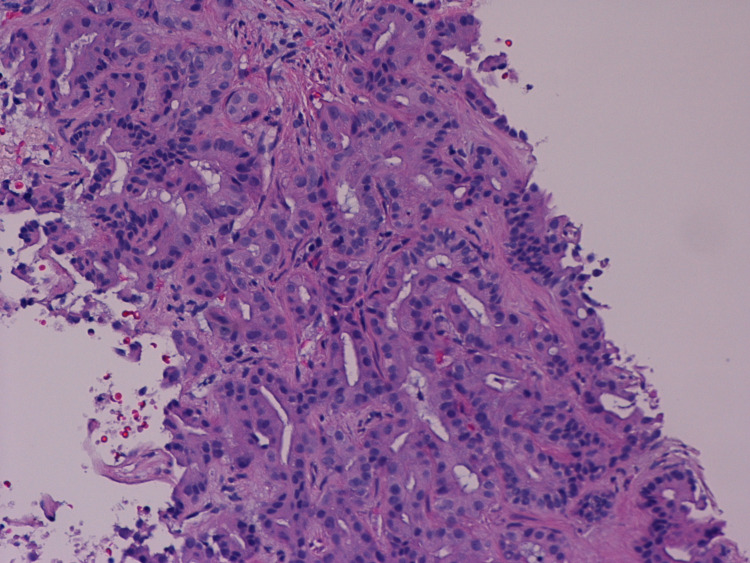
Intrahepatic cholangiocarcinoma, moderately differentiated, with residual gland formation showing foci of architectural irregularity and gland fusion (Hematoxylin/Eosin stain, 400X)

**Figure 3:**
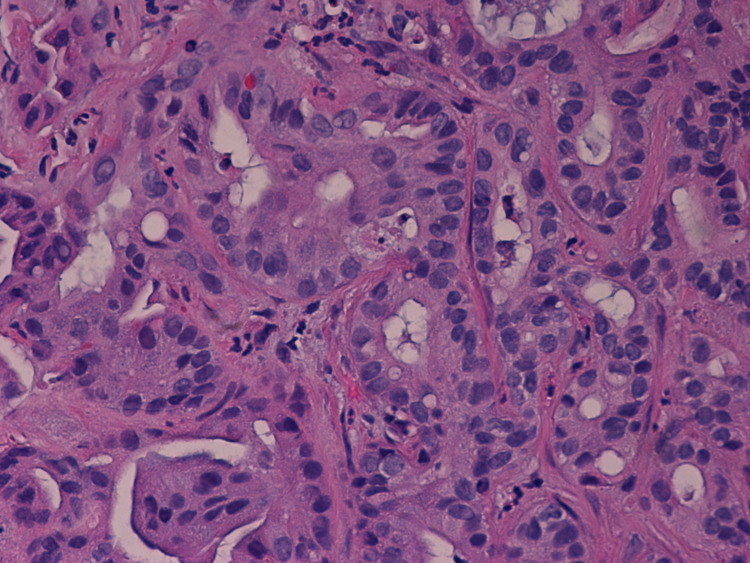
Intrahepatic cholangiocarcinoma, moderately differentiated, showing increased N/C ratio, hyperchromatic nuclei, necrosis, mitotic figures, and nuclear pseudoinclusions (Hematoxylin/Eosin stain, 600X) N/C ratio: Nuclear-cytoplasmic ratio.

**Figure 4:**
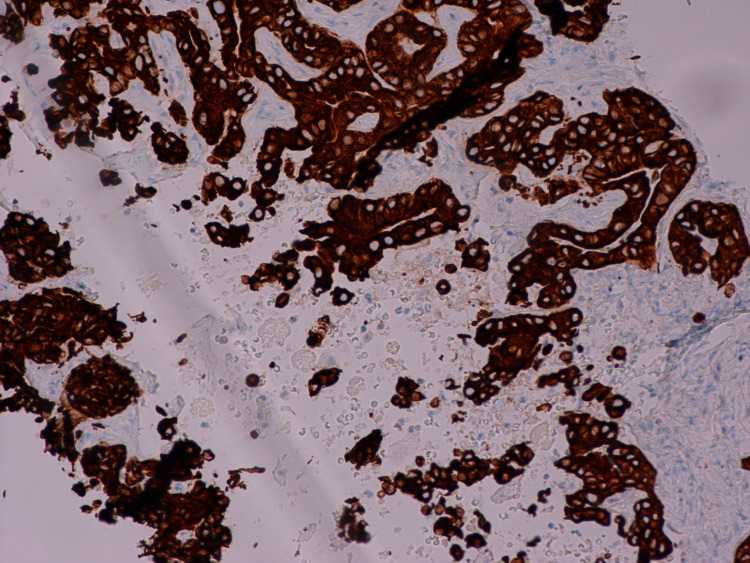
Intrahepatic cholangiocarcinoma, highlighted by a positive Cytokeratin 7 immunohistochemical stain (CONFIRM anti-Cytokeratin 7, SP52, rabbit monoclonal antibody: Ventana/Roche, 400X)

No evidence of other suspicious lymphadenopathy was noted. A positron emission tomography (PET) scan was obtained, which showed uptake in the primary left hepatic biliary lesion. No other areas of abnormal uptake were reported in the liver. The patient underwent a computerized tomography (CT) scan-guided biopsy of the liver mass, which revealed moderately differentiated adenocarcinoma (Figures 2, 3, 4).

**Figure 5:**
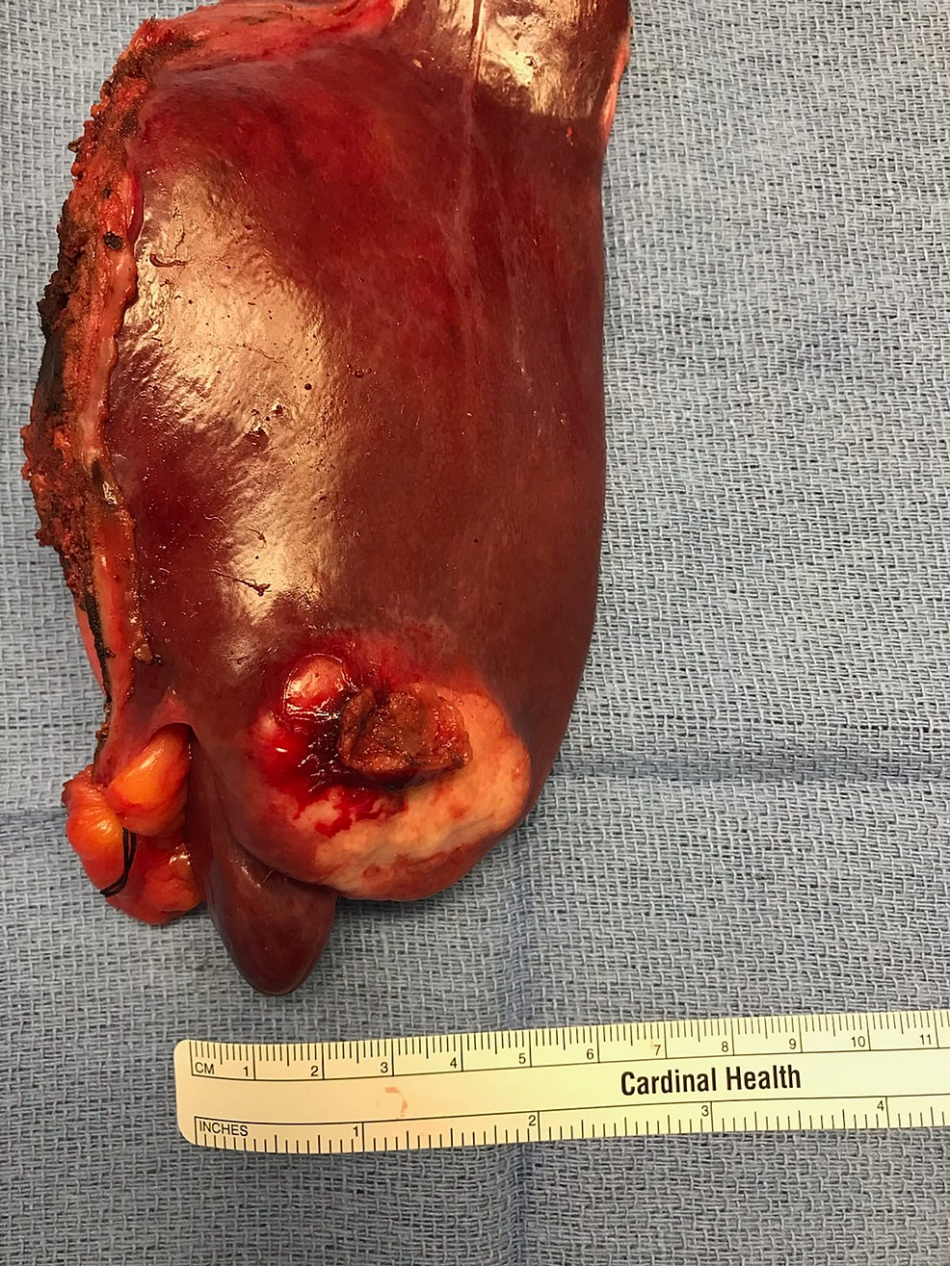
Post-partial hepatectomy showing intraluminal bile ductular dysplasia, proliferation, and multiple nodules of moderately differentiated, intrahepatic cholangiocarcinoma, highlighted in the left lateral section of the liver (segments II and III)

The diagnosis of intrahepatic CCA was made, and the mass was positive for the tumor markers such as CK7, CK19, CK9/18, and CA 19-9. The patient was referred to surgery and underwent partial hepatectomy of the liver segments 2 and 3 (Figure 5). The patient also underwent removal and biopsy of five lymph nodes, specifically the hepatic artery lymph node, hilar lymph node, peripancreatic lymph node, and two periceliac lymph nodes. Metastatic adenocarcinoma was reported only in the hepatic artery lymph node. The patient was referred for genetic testing and received a CustomNext-Cancer gene test. It demonstrated two pathogenic mutations, CHEK2 and MUTYH. She was heterozygous for both the c.1100delC mutation on the CHEK2 gene and c.1227_1228dupGG mutation on the MUTYH gene. No other pathogenic mutation was found on the 91 other genes tested. The patient was started on cisplatin-based chemotherapy and Gemzar and successfully completed her full course of chemotherapy with minimal adverse effects. The results of a repeat PET scan and CT scan of the chest and abdomen did not reveal any evidence of recurrence, and she appears to be in remission [7].

## Discussion

The CHEK2 (checkpoint kinase 2) mutation is inherited in an autosomal dominant pattern and can present with single or multiple cancers simultaneously [8]. The CHEK2 marker signals a serine-threonine kinase, CHK2, to activate the G1/S and G2/M checkpoints during the cell cycle and maintain the functionality of the cells [8]. The most common mutation that is found in the CHEK2 gene is c.1100delC [8,9]. Mutations in CHEK2 have been linked mainly to breast and colorectal carcinoma but also present in gastric, prostate, ovarian, thyroid, lung cancer, melanoma, and glioblastoma [9].

An extensive literature review of Latin American studies on CHEK2 mutation by Guauque-Olarte et al. examined 147 studies of various cancer samples of which 39 studies revealed mutations in CHEK2 [10]. The most common cancer present with a CHEK2 mutation was CCA with 8.6% [10]. Moreover, the MUTYH mutation is associated with the autosomal recessive condition, MUTYH-associated polyposis (MAP), that has been previously linked to CCA [11,12]. The MutY DNA glycosylase (MUTYH) gene undergoes oxidative DNA damage repair via an adenine DNA glycosylase protein by excising improperly placed adenine bases on the DNA backbone [11,12]. Additionally, the DNA glycosylase protein can also activate apoptosis in various cells by inducing single-strand breaks [13,14]. MAP is caused by a c.1227_1228dupGG mutation of the MUTYH gene and is strongly associated with an increased incidence of breast and colorectal cancers [13,14].

In a germline variant analysis study led by Maynard et al., germline mutations were tested in 131 patients with 63.4% and 19% of patients consisting of a diagnosis in intrahepatic CCA and extrahepatic CCA, respectively [15]. Out of the 83 patients with intrahepatic CCA, only 12 patients tested positive for germline mutations, including BRCA1, BRCA2, MUTYH, BAP1, PMS2, and APC [15]. Out of the 21 patients with extrahepatic CCA, only four patients tested positive for germline mutations, including APC, BRCA1, FH, and monoallelic MUTYH [15]. This study was able to show an association of MUTYH with intrahepatic CCA confirmed with a CT-guided biopsy [15]. It is important to identify the sub-group of patients who may be at a higher risk for CCA associated with MUTYH and CHEK2 and also their family members [16]. CT-guided biopsies of cholangiocarcinoma have an increased incidence of introducing very high rates of peritoneal seeding and implicate on disease recurrences [17]. Therefore, CT-guided biopsies and percutaneous fine-needle aspiration (FNA) for extrahepatic and perihilar lesions are generally avoided [17]. However, CT-guided biopsy is needed for tissue diagnosis of large intrahepatic lesions with unspecific imaging findings prior to surgery [17]. Following established screening and early detection guidelines, the first-year survival rate for early-stage cancers can drastically increase, and the affected patient population can participate in treatment-specific clinical trials [16].

## Conclusions

Germline mutations, such as CHEK2 and MUTYH, have been linked separately to CCA. Our case is about a young female patient with CCA who had both CHEK2 and MUTYH mutations. There is limited data on this subject, and further research is needed to explore this association and its impact on the presentation and survival of CCA patients. Testing individuals with CCA and their at-risk family members for CHEK2 and MUTYH mutations could possibly result in earlier diagnosis and effective management protocol.
